# Enterohemorrhagic *Escherichia coli* Detection by Aptamer-Functionalized Stokes-Shifted Quantum Dots

**DOI:** 10.1021/acsomega.6c02442

**Published:** 2026-06-04

**Authors:** Fahd Khalid-Salako, Kaan Tiktaş, Hasan Kurt, Meral Yüce

**Affiliations:** † Faculty of Engineering and Natural Sciences, 52991 Sabanci University, Istanbul 34956, Türkiye; ‡ Sabanci University Nanotechnology Research and Application Center (SUNUM), Istanbul 34956, Türkiye; § Department of Biomedical Engineering, School of Engineering and Natural Sciences, Istanbul Medipol University, Istanbul 34810, Türkiye; ∥ Research Institute for Health Sciences and Technologies (SABITA), Istanbul Medipol University, Istanbul 34810, Türkiye; ⊥ Nanosolar Plasmonics Ltd., Kocaeli 41400, Türkiye

## Abstract

*Escherichia coli* O157:H7 is a prolific serotype of the Shiga toxin-producing *E. coli* strain, known for its enterohemorrhagic pathogenicity and transmission in contaminated food and water. The O157:H7 serotype, evolving from the nonpathogenic sorbitol-fermenting O55:H7 strain, is widely implicated as the causative organism in numerous food poisoning outbreaks reported globally. The public health implications of the spread of this pathogen necessitate its detection through field-deployable sensing systems, reproducible at scale. Accordingly, this study describes the development of a simple aptasensing setup based on magnetically separated Quantum Dots (QDs) with typical Stokes-shifted luminescence, decorated with an *E. coli*-specific aptamer sequence. The QD was covalently functionalized with the aptamer (Apt) by simple carbodiimide chemistry, producing a QD-Apt conjugate, and incubated with a cDNA-MB conjugate (produced by covalently functionalizing magnetic beads (MB) with a ssDNA sequence partially complementary to the aptamer), yielding the QD-Apt@cDNA-MB complex. Displacement of the cDNA-MB conjugate from the complex by cells of the pathogen, with which the QD-Apt binds with higher affinity, led to the loss of luminescence intensity in the magnetically separated supernatant containing the remaining QD-Apt@cDNA-MB construct (alongside the displaced cDNA-MB fragments), constituting the signal transduction mechanism of the *E. coli* detection system. The luminescent biosensor achieved a low detection limit (∼56 CFU · mL^–1^), high sensitivity, and specificity for the target pathogen, against other common food pathogens. The performance of the luminescent biosensor, in the absence of prior enrichment or sample concentration steps, renders it promising for large-scale environmental monitoring applications, toward the control of *E. coli* O157:H7 contamination.

## Graphical abstract

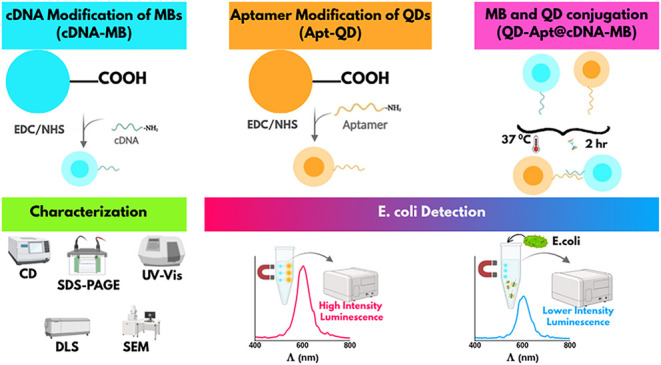

## Introduction

Quantum Dots (QDs) are zero-dimensional nanomaterials typically fabricated by bottom-up “hot injection” techniques with precise final size control.^1,2^ QDs are generally semiconductor nanocrystals with size-dependent quantized energy levels, resulting in interesting optical and electronic properties.^3,4^ While photoluminescence in bulk semiconductor materials is governed by material-specific band gaps and emission wavelengths, the quantum confinement effect arising in QDs leads to tunable energy band gaps.^5,6^ It is important to note that quantum confinement arises as these semiconductor crystals transition from the bulk materials to nanomaterials approaching the de-Broglie wavelength of electrons (and more specifically, the Bohr radius of the semiconductor material-specific exciton), causing a constriction of excitons (in all dimensions in QDs).^7^ Invariably, the emission profiles of QDs are heavily influenced by their sizes and consequent exciton decay behavior. Additionally, QDs exhibit broad excitation/absorption wavelengths and narrow, Stokes-shifted emission spectra.^8^ This enables QDs to be used for a wide range of biosensing and imaging applications where their tunable sizes can be exploited for contrasting and multiplexing purposes, with broadband excitation light sources.^7,9^

The optical properties of QDs have been exploited over time for various biomedical purposes, especially in diagnostics.^10^ At a fundamental level, some applications involve the biotagging of QDs with finely tuned, predetermined sizes and emission spectra for the direct detection of bioconjugate analytes under broadband illumination.^11,12^ However, more sophisticated bioassays have been developed, with exciton generation from luminescent biomolecules and chemical entities, or electrical input from redox complexes, and signal readout in the form of photoluminescence, photocurrent, and electrochemiluminescence, among others.^7^ QDs have also been used in biosensing systems based on “fluorescence quenching,” when a quencher, through several mechanisms, diminishes fluorescence in close proximity to the biofunctionalized QDs within the assay media.^9^ This, for example, is the operating principle behind the widely used Förster resonance energy transfer (FRET) biosensors, where the QD emission bands overlap with the quenchers’ absorption spectra.^13–15^ These are some of the concepts that have led to the application of QDs in optical biosensors for the detection of human immunoglobulin G and *Escherichia coli* bacteria,^16^ heavy metals,^17^ and serum interleukins,^18^ among several other disease biomarkers and environmental contaminants of interest. Beyond biosensing, QDs have also found use in bioimaging;^19^ photodynamic therapy for cancers^20^ and COVID-19;^21^ and in traceable/targeted drug delivery;^22,23^ among several other relevant biomedical applications.

*E. coli* is a commensal bacterium found as part of the normal human intestinal flora, and which has even been adopted clinically as a probiotic for certain intestinal issues.^24^ However, *E. coli* has also demonstrated significant pathogenicity in immunocompromised people.^25^ The high mutation rate and horizontal gene transfer propensity of *E. coli* have led to the generation of several subtypes implicated in systemic infections, including uropathogenic, enteropathogenic, enterohemorrhagic, enterotoxigenic, enteroaggregative, and diffusely adherent *E. coli*, among others.^26^ One of the most prolific enterohemorrhagic strains is *E. coli* O157:H7, a food pathogen that has been reported to cause infectious disease outbreaks due to contaminated food and water.^27,28^

The public health and safety implications of *E. coli* O157:H7 contamination in food and water in the environment necessitate its early detection to reduce the incidence of outbreaks and associated health and economic effects.^29^ Traditional bacterial detection methods still prevalent, particularly in clinical practice, rely on bacterial culture, enzyme-linked immunoassays, polymerase chain reactions, and loop-mediated isothermal amplification techniques, which ultimately impose time, cost, and expertise limitations, precluding their adaptation for point-of-care use.^30^ In response, several innovative biosensor systems based on fluorescence,^27^ and electrochemical^31^ signal transduction phenomena, among others, have been reported in the literature. Optical biosensing of *E. coli* on the basis of QD luminescence has severally been reported in recent literature. Kumari and Chaudhary^32^ developed a “turn-off” sensing system for the selective detection of *E. coli* with carbon dots derived from recycled plastic matter. Similarly, Pandit et al. reported an aptamer-decorated QDs biosensing system, achieving detection limits impressively down to 100 cells of *E. coli*. Other QD-based systems based on sandwich immunoassays have also recorded similar detection limits like 20–50 CFU · mL^–1^.^33^ The operational requirements of a field-deployable biosensor system render them more amenable to aptamer decoration rather than antibodies, due to the stringent storage conditions and stability limitations of antibody molecules as biorecognition elements.^34,35^ Additionally, the development of a scalable detection system for rapid point-of-care detection of *E. coli* and other bacterial pathogens in environmental monitoring contexts requires that such systems consist of simple components that can be reproducibly fabricated and functionalized at scale. Accordingly, this research reports a luminescent *E. coli* O157:H7 (EC) detector, using EC aptamer-functionalized down-converting QDs.

## Results and Discussion

The biosensing principle of the aptamer-functionalized QD (QD-Apt) platform relies on the down-converting fluorescent properties of QDs, combined with aptamer specificity for the EC cell surface, based on their tertiary spatial configurations.^36^ The aptamer sequence used was obtained from Wu et al.^37^ Generally, the Stokes-shifted emission of QDs serves as the biosensing platform’s signal transduction mechanism, while biofunctionalized magnetic beads (MBs), designed to bind the anti-EC aptamer with lower affinity than the primary target, enable the separation of unbound QD-Apt conjugates for both purification and bioassay purposes. Generally, the QD-Apt conjugate was incubated with partially complementary DNA-functionalized MB (cDNA-MB) to derive the QD-Apt@cDNA-MB complex. When a magnetic field is applied, the complex provides strong luminescence at ∼600 nm following 325 nm excitation. When the QD-Apt@cDNA-MB construct is incubated with EC, the pathogen binds QD-Apt, displacing the relatively weakly bound cDNA-MB component, diminishing the luminescence intensity on signal acquisition. Similar biosensing techniques have been reported in the literature for the detection of various food pathogens.^38,39^ A schematic of the EC detection mechanism adopted in this study is presented in Scheme 1.

**Scheme 1:**
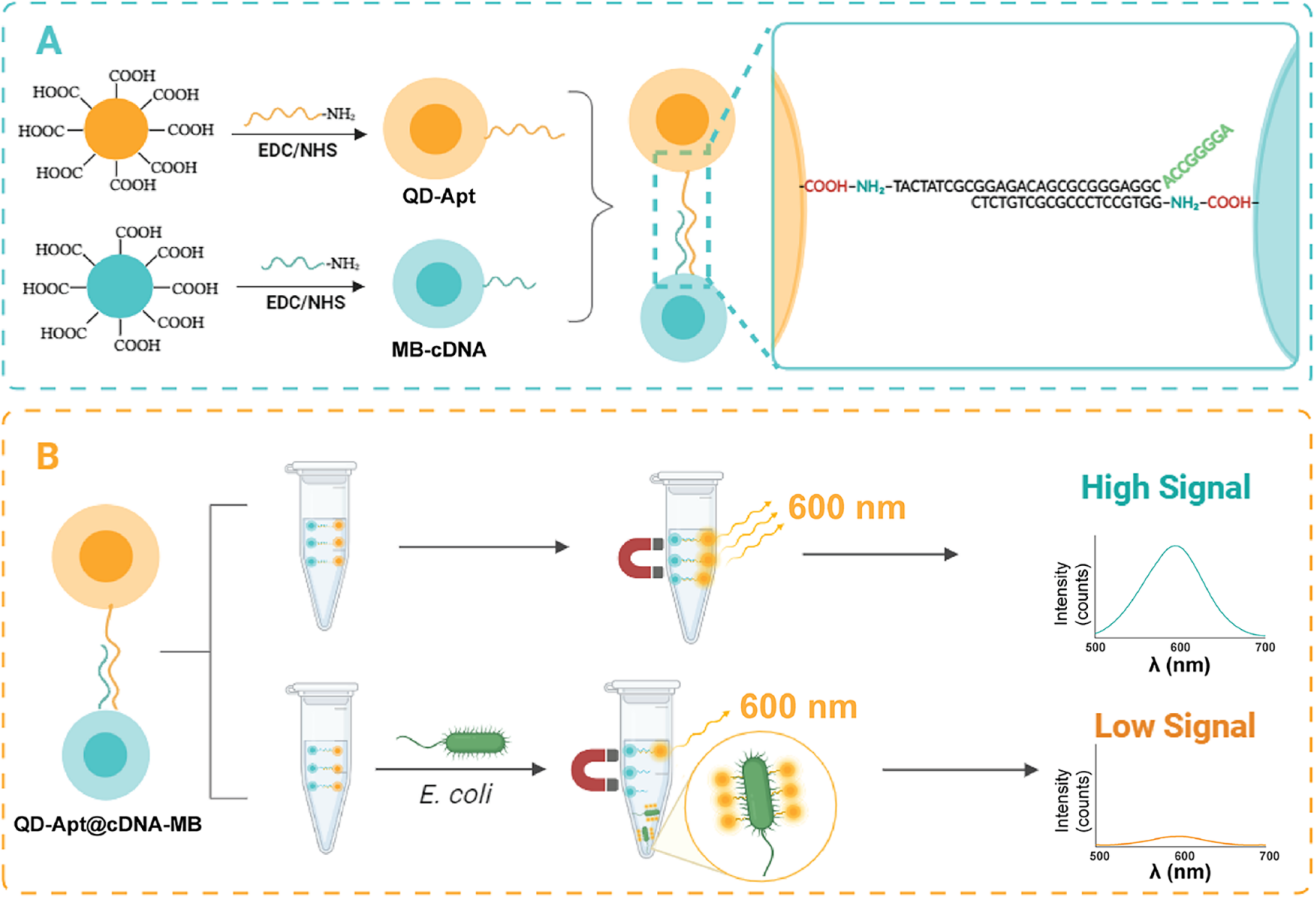
A Schematic of the *E. coli* Detection Mechanism by the QD-Based Luminescent Platform, Including (A) A Workflow of the ssDNA Decoration of the NPs; and (B) the Excitation and Signal Transduction Mechanism of the QD-Apt@cDNA-MB System

### Biofunctionalization and Characterization

Results of characterization studies confirming the biofunctionalization of the QDs are presented in Figure 1, while MB characterization studies are presented in Figure 2. The carboxylated QD and MB were functionalized by amine coupling through carbodiimide chemistry, where free surface carboxyl groups were activated with EDC and sulfo-NHS, after which incubation with the anti-EC aptamer and EC cDNA resulted in the formation of amide bonds between the QD/MB and the terminal amine groups of the respective oligonucleotides, as illustrated in Figure 1A. To confirm the successful functionalization of the QD and MB, characterization experiments were conducted.

**Figure 1:**
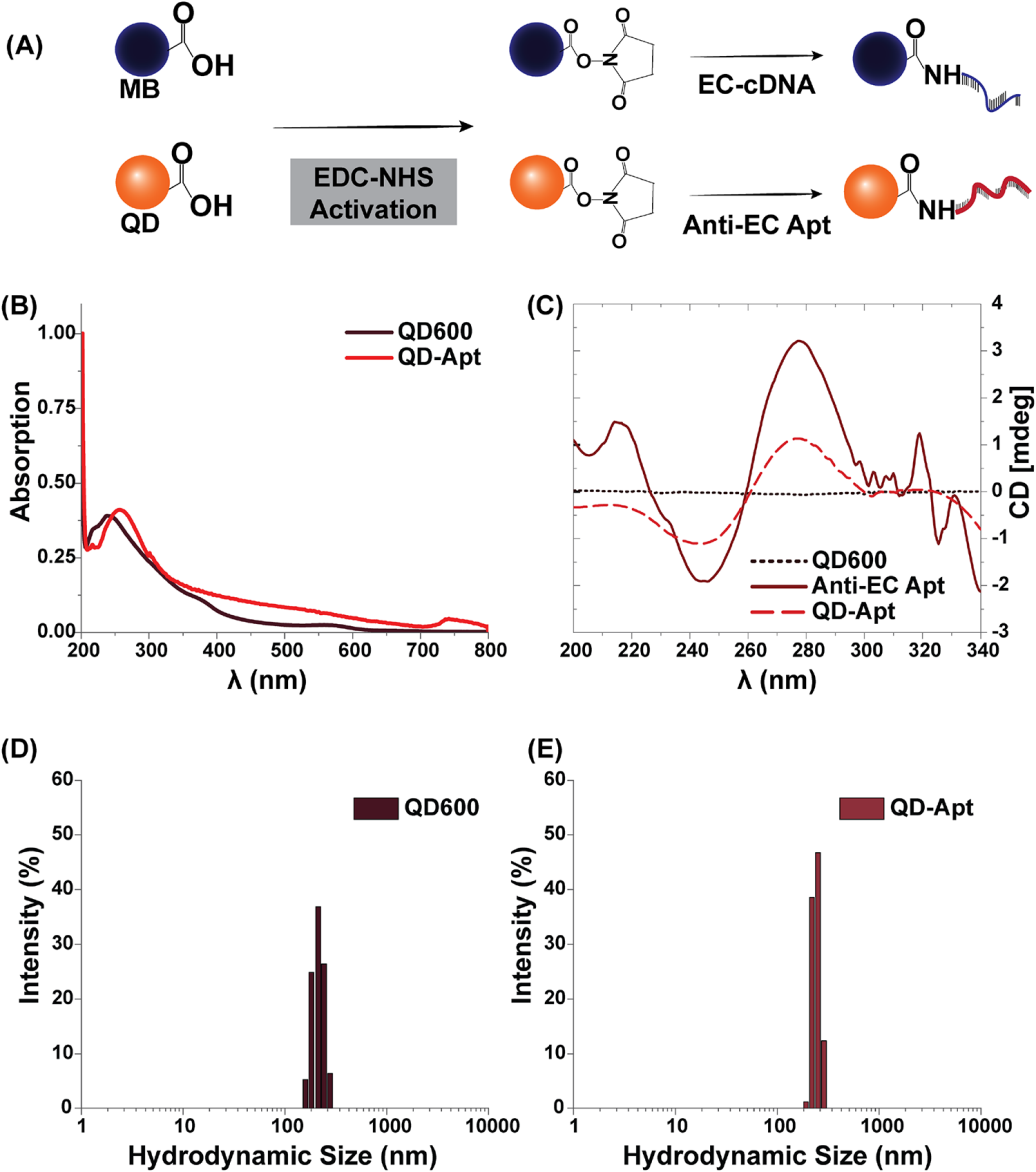
QD characterization studies, showing: (A) an illustration of the biofunctionalization workflow, including the EDC-NHS activation of the carboxylated QD and MB surfaces and incubation with the respective oligonucleotide sequences; (B) the UV–vis absorption spectra of the QD (QD600) and the QD-Apt conjugate; (C) the CD spectra of the QD, the anti-EC Apt, and the QD-Apt conjugate; and (D and E) hydrodynamic size distributions of the QD and QD-Apt respectively, obtained by DLS.

**Figure 2:**
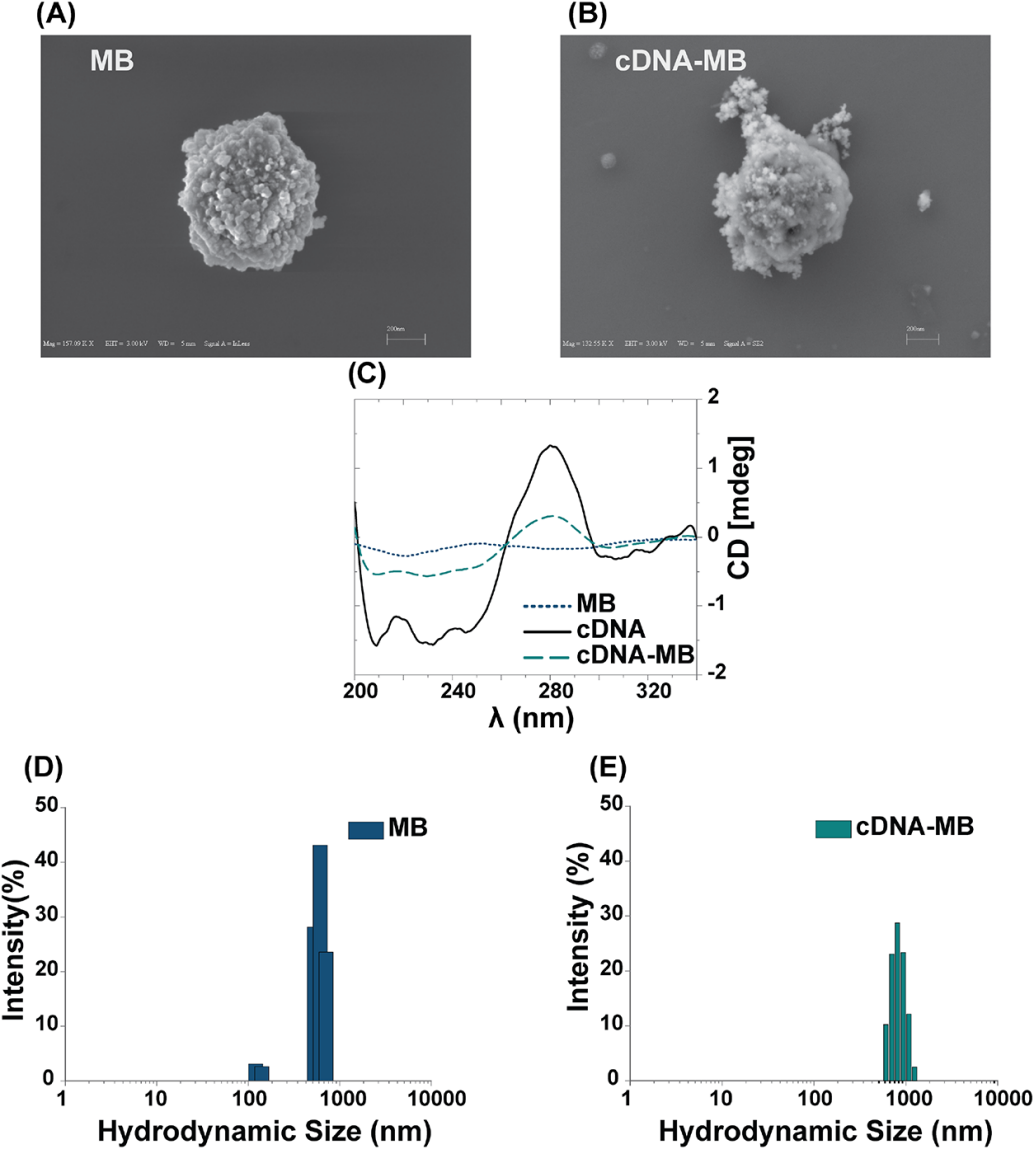
MB characterization studies, showing: (A and B) secondary electron images of the MB and cDNA-MB conjugate (scale bars correspond to 200 nm); (C) the CD spectra of the MB, cDNA, and the cDNA-MB conjugate; and (D and E) hydrodynamic size distributions of the MB and cDNA-MB conjugate, respectively, obtained by DLS.

From the UV–vis absorption profiles of the modified QD, the broad QD600 absorption peak observed at ∼242 nm was slightly red-shifted, suggesting oligonucleotide presence, given the well-documented optical activity of DNA bases in the 200–300 nm spectral region.^40^ Further confirmation of successful oligonucleotide decoration of the QD and MB was provided by the CD spectroscopic studies. While CD spectroscopy typically characterizes the secondary and tertiary structures of polypeptides and proteins, such in-depth insights were not the focus of the current study. Simply, the chirality of oligonucleotide molecules, resultant differential absorption of L- and R- circularly polarized light, and consequent reflection of elliptically polarized light;^41,42^ a phenomenon absent in the bare QD and MB nanoparticles (NPs), provides a conclusive basis for the presence of oligonucleotide sequences on the material surfaces. Accordingly, we obtained CD spectra of the oligonucleotides, QD/MB, and the QD-Apt/MB-cDNA conjugates separately, in the UV region, where electronic transitions are representative of secondary and tertiary structures resulting from oligonucleotide folding. As shown in Figures 1C and 2C, both the aptamer (Anti-EC Apt) and complementary DNA (cDNA) oligonucleotides expectedly recorded increased ellipticity of the reflected light’s polarization at ∼220 nm, representative of an *n*π* electronic transition, with several other ellipticity peaks in the near UV region, suggestive of tertiary structure-related transitions.^41^ In contrast, the bare QD and MB samples showed no significant changes in the circularly polarized light, with significantly increased ellipticity in corresponding spectral regions observed after the biofunctionalization procedures to produce QD-Apt and cDNA-MB conjugates (Figures 1 and 2).

Additionally, the QD and MB nanoparticles were observed to have increased hydrodynamic radii and corresponding narrower size distributions after biofunctionalization, attributable to the incorporation of water molecules around the oligonucleotide-decorated NP complexes (Figures 1D,E, and 2D,E). The hydrophilicity of oligonucleotide molecules forms a hydrated shell around the functionalized NPs.^43^ As a result, the average hydrodynamic radius of the QD increased from 213.94 nm (QD600) to 239.82 nm (QD-Apt), while that of the MB increased from 587.5 nm (MB) to 837.36 nm (cDNA-MB). Notably, the bare QDs are expected to have actual sizes ∼3–4 nm.^44^ Therefore, while aptamer decoration is indicated by the observed changes in hydrodynamic sizes, the markedly high sizes recorded (∼200 nm)­can be attributed to agglomerations within the colloidal solutions measured by DLS,^45,46^ as well as the over-representation of these agglomerates in the size-distribution measurement, given the low DLS sensitivity to smaller particles (<10 nm).^47^

To further ascertain the success of the biofunctionalization step, PAGE analysis was conducted, including the oligonucleotide samples; respective QD-Apt and cDNA-MB conjugates; and the QD-Apt@cDNA-MB complex, as presented in Figure 3. Following extensive washing steps, the QD-Apt, cDNA-MB, and QD-Apt@cDNA-MB samples did not show the PAGE bands observable with the unbound oligonucleotide samples. This implies that the biofunctionalized NP samples were free of unbound DNA molecules, and findings from the CD and UV–vis studies emanated from DNA molecules stably, covalently bound to the NP surfaces, conclusively demonstrating the successful oligonucleotide decoration of the QD and MB NPs.

**Figure 3:**
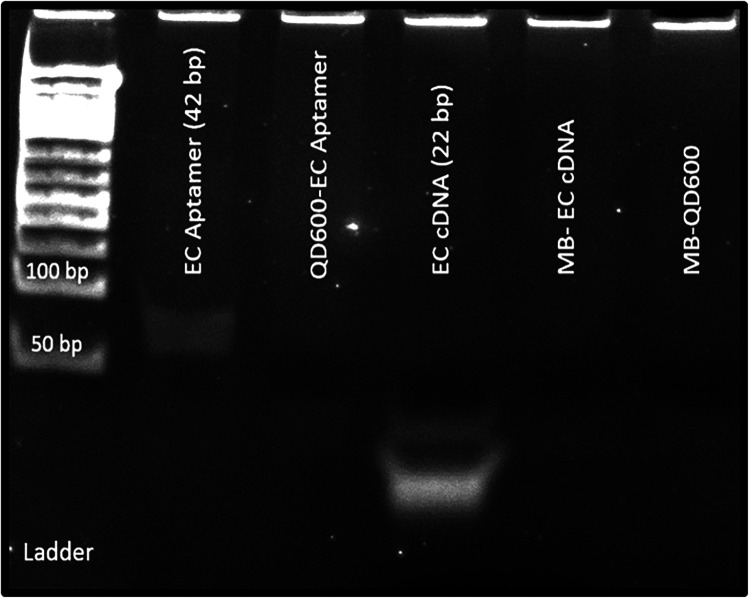
Polyacrylamide gel electrophoresis (PAGE) bands of the unbound aptamer (EC aptamer); the QD-Apt conjugate (QD600-EC aptamer); the unbound cDNA (EC cDNA); the cDNA-MB conjugate (MB-EC cDNA); and the QD-Apt@cDNA-MB complex (MB-QD600).

### Bacterial Detection and Sensing Performance

#### Bacterial Culture

After transferring a single colony of the overnight *E. coli* culture to the NB medium, the kinetic growth pattern of the pathogen was monitored by recording optical density measurements at 600 nm, computing the concentration at hourly intervals over a 10 h period and subsequently at bi-hourly intervals over the next 10 h period (Figure 4). The recorded growth pattern of the pathogen indicates a maximal concentration at 12 h, informing the design of subsequent biosensing performance studies and cell passaging schedules. To check the culture for contamination, light microscopy imaging was done, following simple staining. Scanning electron microscopy studies were also conducted; in addition to the temporal growth kinetics, light microscopy images, as well as the secondary electron images of the EC culture, are presented in Figure 4.

**Figure 4:**
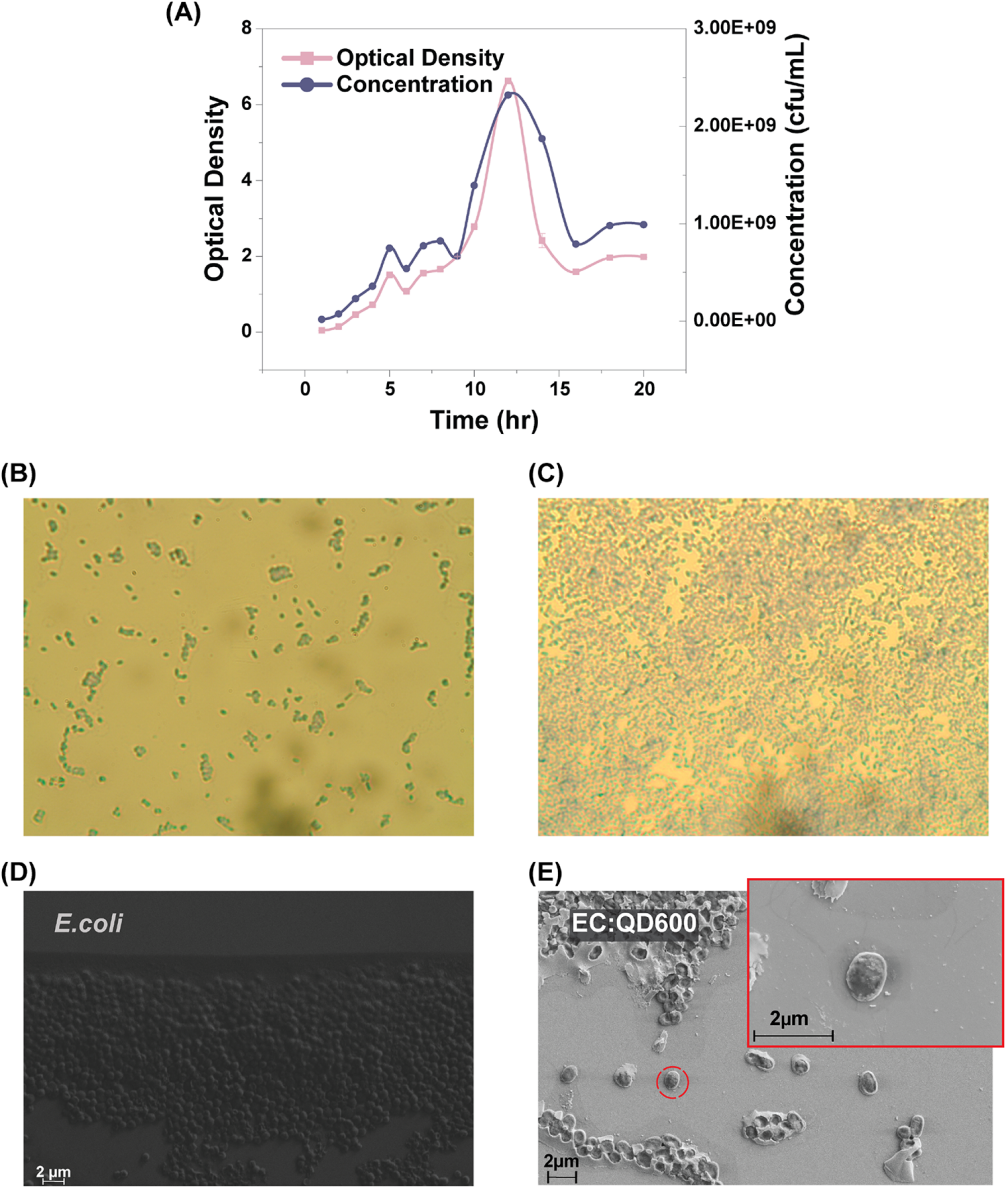
*E. coli* culture characterization studies, including: (A) temporal growth patterns of the *E. coli* culture, monitored in terms of optical density (OD) and concentration (CFU · mL^–1^); (B and C) light microscopic images of the EC culture, and secondary electron images of (D) the *E. coli* cells, obtained at 7.5k× magnification and 2 kV accelerating voltage (EHT); and (E) the cells after incubation with the QD-Apt conjugate, obtained from an in-lens secondary electron detector at 50k× magnification and 2 kV EHT.

#### Biosensing Sensitivity and Specificity

For the EC detection bioassays, the QD-Apt@cDNA-MB complex was incubated with the EC solutions prepared by pelletizing the EC culture (through centrifugation) and replacing the supernatant with PBS buffer. Incubation with EC was done for 30 min, after which a magnetic field was applied for separation, and the magnetic supernatant was optically measured, exciting unbound QD-based complexes. The entire signal acquisition procedure, including QD-Apt@cDNA-MB incubation with the analyte (30 min), magnetic separation, and optical measurement, typically lasted about 45 min in total per sample. The signal transduction mechanism of the QD-based EC biosensing platform was based on the loss of luminescence/fluorescence intensity in the magnetically separated supernatant of the mixture. The biosensing performance of the QD-Apt@cDNA-MB platform was then assessed by incubating the NP complex with EC at a logarithmic series of concentrations up to 10^6^ CFU · mL^–1^. Incubation of the QD-Apt@cDNA-MB complex with blank PBS buffer served as the 0 CFU · mL^–1^ sample and blank reference. Expectedly, luminescence intensity (measured as the peak height of the emission peak at ∼600 nm) decreased with increasing EC concentration, as more EC cells displaced the cDNA-MB conjugate from QDs, diminishing the number of unbound QDs that could be separated and optically excited. The signal intensity at each concentration was computed by

1 $$\Delta I=I_{0}-I$$

where *I*_0_ is the luminescence intensity of the blank reference and *I* is the obtained luminescence at each concentration. A linear calibration curve was obtained by plotting Δ*I* against log_10_ concentration. Detection limits were calculated based on literature-established protocols as^48,49^

2 $$\Delta I_{\mathrm{LOB}}=\Delta I_{0}+\left(1.65\times \sigma \right)$$

3 $$\Delta I_{\mathrm{LOD}}=\Delta I_{\mathrm{LOB}}+\left(1.65\times \sigma \right)=\Delta I_{0}+\left(3.33\times \sigma \right)$$

where the 0 CFU · mL^–1^ sample was adopted as the blank sample, with Δ*I*_0_ = *I*_0_–*I*_0_ = 0; similarly, σ, which quantifies the noise of the luminescence setup, is calculated as the standard deviation of measurements obtained with 0 CFU · mL^–1^ pathogen incubation (*n* = 11). Accordingly, Δ*I*_LOB_ and Δ*I*_LOD_ are signal intensity values from which the limits of blank (LOB) and detection (LOD) can be obtained as corresponding concentration values on the linear regression curve (Figure 5B). A σ value of 130.7 was obtained, and the calculated Δ*I*_LOD_ = 435.22 corresponds to a 10^1.75^ = 56.23 CFU · mL^–1^ LOD value on the basis of the linear regression equation of the calibration curve (Figure 5B).

**Figure 5:**
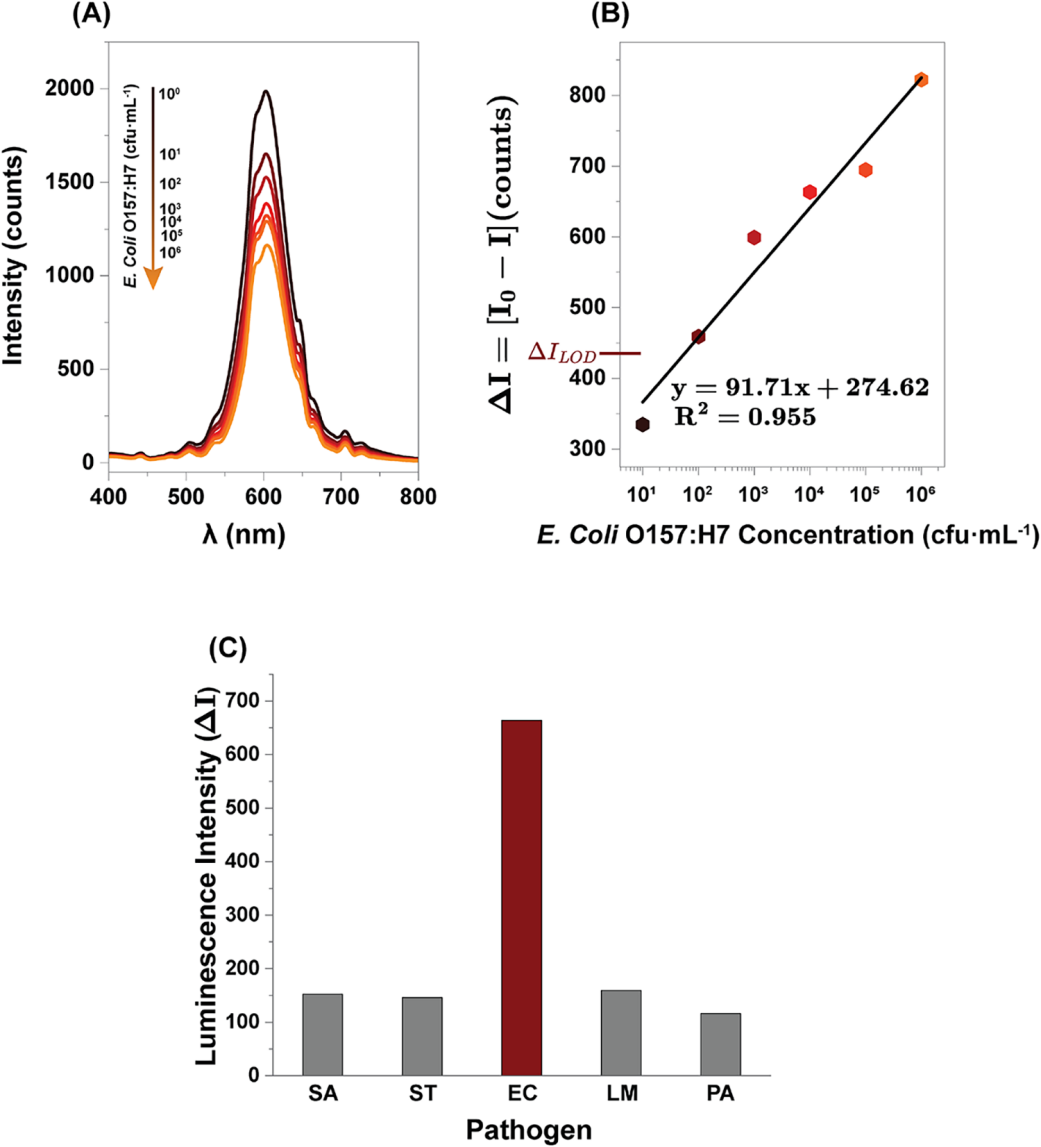
Sensitivity and specificity findings, including: (A) emission spectra obtained from the various EC concentrations; (B) the linear calibration curve of Δ*I* against log_10_ concentration. The equation of the line is indicated, as well as the goodness-of-fit (*R* ^2^) value; and (C) signal intensities obtained with 10^4^ CFU · mL^–1^ of SA, ST, EC, LM, and PA cultures.

The specificity of the EC-sensing construct was also assessed by incubating the complex with other common pathogens, including *Staphylococcus aureus* ATCC 29213 (SA); *Salmonella typhimurium* ATCC 14028 (ST); *Listeria monocytogenes* ATCC 19115 (LM); and *Pseudomonas aeruginosa* ATCC 27853 (PA), all at 10^4^ CFU · mL^–1^, comparing the signal intensities obtained against those obtained from EC at the same concentration. Findings of both sensitivity and specificity bioassays are presented in Figure 5.

The luminescent EC biosensor demonstrated acceptable linearity over the concentration range explored in this study (*R* ^2^ = 0.955), with a detection limit ≈56 CFU · mL^–1^; and a sensitivity of 91.71 counts increase in Δ*I* for each log_10_ unit increase in CFU · mL^–1^, providing comparable performance with recently reported platforms for the optical detection of *E. coli* O157:H7.^33,50,51^ Some other fluorescent biosensor setups developed for the detection of *E. coli* have reported similar detection limits, ranging from 0.05 to 420 CFU · mL^–1^, albeit with multiple rounds of PCR amplification.^52–55^ Similarly, Bai et al. reported a carbon QDs-based fluorescence aptasensing of *E. coli* based on the EC concentration-dependent attenuation of Ag NPs absorption and quenching of the CQDs emission, reaching a detection limit ∼185 CFU · mL^–1^.^56^ Similarly, Ling et al. recently reported a magnetic NPs and carbon QDs-based fluorescent biosensing platform for *E. coli* O157:H7 detection, based on a monoclonal antibody biorecognition element, achieving detection limits as low as ∼25 CFU · mL^–1^. A concise overview of similar luminescent biosensing platforms for *E. coli* detection is presented in Table 1.

**Table 1:** An Overview of Some Fluorescent/Luminescent Nanoparticle-Based *E. coli* Detection Platforms Reported in the Literature over the Past Five Years

**pathogen strain**	**detection mechanism**	**bioconjugate**	**LOD**	**refs**
EC O157:H7	fluorescent CD release from BON	boronic acid capture probes and monoclonal antibody detection probes	25 CFU · mL^–1^	^57^
	enhanced CD fluorescence by EC-attenuation of Ag NPs quenching	aptamers	185 CFU · mL^–1^	^56^
EC O157:H7	MNP-conjugated CD fluorescence attenuation following EC-Apt binding and MNP displacement	aptamer	487 CFU · mL^–1^	^51^
EC O157:H7	displacement of cDNA from the EC Apt, triggering a cascade SDA process and fluorescent ThT-containing G-quadruplex production	aptamer	10 CFU · mL^–1^	^58^
EC O157:H7	fluorescence of magnetically separated antibody-decorated CD-microspheres bound to EC cells	antibody	240 CFU · mL^–1^	^59^
EC CICC 21530	CD-Apt complex binding of EC captured on a magnetic composite material with fluorescence quenching and photothermal sterilization	aptamer	2 CFU · mL^–1^	^60^
EC O157:H7	a Zr-MOF binding of antibody-functionalized MNPs-captured EC cells, with Zr-MOF fluorescence following H_2_O_2_ addition	antibody	1 CFU · mL^–1^	^61^
EC O157:H7	LFIA-based detection of EC by aggregation-induced luminescent NPs	antibodies	306 CFU · mL^–1^	^62^
EC O157:H7	bacterial quenching of silica NPs’ fluorescence	antibody	160 CFU · mL^–1^	^63^
EC O157:H7	aptamer-decorated QD detection of magnetically separated EC cells	capture and detection of aptamer probes	100 CFU	^64^
EC O157:H7	fluorescence of EC-bound Zr-MOF in the presence of Al^3+^	direct MOF binding	1000 CFU · mL^–1^	^65^
EC O157:H7	restoration of CD fluorescence after displacement from a complex with a COF fluorescence acceptor	antibody	7 CFU · mL^–1^	^66^
EC O157:H7	fluorescence of silica-bound QD probes by LFA	antibody	50 cells · mL^–1^	^67^
	fluorescent tetraphenylethylene binding of magnetically separated iron oxide-MIP core–shell MNPs	capture MIPs and detection aptamer	100 CFU · mL^–1^	^68^
EC O157:H7	loss of fluorescence in the magnetically separated reaction supernatant, following QD displacement from MNPs by bacterial cells	aptamer	56 CFU · mL^–1^	^a^

a This work: BON: breakable organosilica nanocapsules; CD: carbon dots; CFU: colony-forming units; COF: covalent organic framework; EC: *E. coli*; LFA: lateral flow assay; LFIA: lateral flow immunoassay; MIP: molecularly imprinted polymers; MNP: magnetic nanoparticles; MOF: metal–organic framework; SDA: strand displacement amplification; and ThT: thioflavin-T.

The specificity of the QD-Apt@cDNA-MB biosensor for EC is demonstrated in Figure 5C, attributable to the highly specific binding of the ssDNA oligonucleotide to the EC cells, as well as the inactivation of active chemical moieties on the NPs with ethanolamine during the biofunctionalization steps, preventing promiscuous binding. The low signal variability observed in the blank buffer measurement (RSD = 6.6%), together with a negligible luminescence response in the presence of nontarget microorganisms, confirms that background-induced quenching is minimal and that the measured signal modulation is governed by specific aptamer–target interactions rather than nonspecific environmental effects.

## Conclusion

Drawing from the significant public health concerns emanating from food contamination with the Shiga toxin-producing *E. coli* O157:H7, this study presents the development of a simple optical biosensing platform capable of sensitive and specific pathogen detection. The detection limits and sensitivity realized without enrichment or sample concentration demonstrate the luminescent platform’s applicability for field operations and high-throughput environmental monitoring projects.

A key novelty of this work lies in the design and implementation of a luminescent sensing platform (QD-Apt@cDNA-MB), which combines the photostability and tunable emission properties of QDs with the specificity and robustness of the oligonucleotide-based biorecognition. This system, in contrast to antibody-based biosensing platforms, provides enhanced biosensor stability and reproducibility under a wide range of operating conditions. Signal transduction with luminescence modulation based on established hybridization dynamics, including cDNA-Apt binding and competitive displacement by EC, also enables sensitive detection with minimal reliance on complex labeling strategies.

The specificity of the system for the target pathogen, alongside fluorescent spectral purity, further supports its potential for multiplexed detection of an array of food pathogens based on QDs with tunable emission wavelengths, biofunctionalized with carefully selected target-specific oligonucleotide sequences. While the present study focuses on buffer-based validation, it is important to note that closely related QD-MB sensing platforms have demonstrated high recovery rates (92–97%) for pathogen detection in complex food matrices in prior studies.^14,69^ Given the shared sensing architecture and signal transduction mechanism, these findings support the expected applicability of the current system in real sample environments. Nevertheless, a comprehensive evaluation in diverse and complex food matrices, alongside system miniaturization for point-of-need deployment, remains an important direction for future work.

## Experimental Section

### Materials and Instrumentation

Carboxylated CdTe QDs 600 ± 5 nm were procured from PlasmaChem GmbH (Berlin, Germany), while carboxylated magnetic beads (MB MHP-800) were purchased from Ocean NanoTech (San Diego, CA, USA). An Anti-EC single-stranded DNA aptamer^37^ and a partially complementary cDNA sequence, both with amine-modified 5′ terminals, were procured from Integrated DNA Technologies (IDT DNA, USA).

Buffer solutions were prepared in-house with chemical consumables purchased from Sigma-Aldrich Chemie GmbH (USA). Other chemical reagents, including the *N*-(3-(dimethylamino)­propyl)-*N*-ethyl carbodiimide hydrochloride (EDC) and an *N*-hydroxy-sulfosuccinimide sodium salt (NHS), were also purchased from Sigma-Aldrich Chemie GmbH (USA).

The fluorescence spectroscopy setup included 325 nm excitation with a Kimmon IK3023R-BR 325 nm 2 mW He–Cd gas laser (Kimmon Koha Co., Tokyo, Japan) and an Ocean Optics USB2000+ preconfigured spectrometer (Ocean Optics, Dunedin, FL, USA).

### Biofunctionalization

The magnetic beads (MB) were functionalized with the cDNA sequence (MB-cDNA), while the QDs were functionalized with the anti-EC ssDNA aptamer (QD-Apt). Since both MBs and QDs were carboxylated, covalent functionalization with the respective oligonucleotide sequences was done by the amine coupling approach. The anti-EC aptamer and cDNA oligonucleotides obtained in powder form were dissolved in sterile distilled water to final concentrations of 100 μM, after which the solutions were heated to 95 °C and immediately cooled on ice to room temperature in order to denature any 3D structures and return the molecules to their specific minimum energy level structures. The carboxylic acid groups on the MB and QD surfaces were activated with EDC (10 mg L^–1^) in MES (2-(4-morpholino)­ethanesulfonic acid) buffer. Subsequently, sulfo-NHS (2 mg L^–1^) and the oligonucleotide solutions (apt to QD; cDNA to MB) were added to the respective media and incubated overnight. Unbound aptamers in the QD-Apt medium were centrifuged to remove unbound aptamer molecules, while a professional magnetic unit was used to separate unbound cDNA molecules from the MB-cDNA medium.

After washing the biodecorated surfaces with sterile distilled water and transferring them to PBS (phosphate-buffered saline), both QD-Apt and MB-cDNA were conjugated. The QD-Apt was incubated with MB-cDNA in PBS buffer for approximately 2 h at 37 °C. A 3-fold excess concentration of QD-Apt solution relative to the MB-cDNA was used to fully saturate the magnetic bead surfaces. A magnetic field was used to remove the unbound molecules from the medium.

### Characterization

The successful functionalization of the MB and QD with cDNA and Apt, respectively, was confirmed by different characterization techniques, including polyacrylamide gel electrophoresis (PAGE); UV–vis-NIR spectroscopy; circular dichroism (CD) spectroscopy; and dynamic light scattering (DLS).

PAGE analyses of the EC aptamer, the QD-Apt construct, the EC aptamer complementary cDNA, the MB-cDNA construct, and the QD-Apt@cDNA-MB conjugate were done on a 6% native PAGE gel and run at 80–120 V. Visualization was done under UV illumination using the Bio-Rad Gel Doc EZ digital system (Bio-Rad Laboratories Inc., USA), following ethidium bromide staining.

The absorption spectrum of QD-Apt was measured using a UV–vis-NIR spectrophotometer (Shimadzu UV-3150, Kyoto, Japan), in the 200–300 nm range; the characteristic absorption bands of DNA bases.^40^ 100×-diluted samples in PBS buffer were measured in quartz cuvettes (Quartz-Suprasil, Hellma GmbH & Co. KG, Mulheim, Germany) against a PBS blank control to reduce background noise.

The differential absorption of circularly polarized light, characteristic of oligonucleotides’ secondary and tertiary structures and chirality, was measured as a basis for the detection of DNA binding on the nonchiral QD and MB surfaces. CD spectroscopy was conducted under a continuous *N*_2_ flow (J-815, Jasco International Co., Tokyo, Japan). Sample solutions in PBS buffer were scanned in CD cuvettes (1 mm light path) at an approximately 100 nm/min scan rate. A blank PBS solution was used as a background control.

Changes in hydrodynamic sizes and size distributions of QDs before and after functionalization were evaluated by DLS (Malvern Zetasizer Nano ZS, Malvern Instruments, Worcestershire, UK), integrated with a vertically polarized He–Ne laser (λ = 633 nm), and at 173°. Samples were diluted 1000× in distilled water, and measurements were conducted in disposable sterile cuvettes. DLS results were visualized with the NNLS algorithm-based device software.

### *E. coli* Detection

The *E. coli* O157:H7 culture (ATCC 25922) in nutrient agar (NA) was incubated overnight at 37 °C, after which a single colony inoculum was transferred to a nutrient broth (NB) medium. The growth pattern of the EC culture in NB was monitored by time-resolved optical density measurements at 600 nm with a Bio-Rad Smart Spec Plus spectrophotometer (Bio-Rad Laboratories Inc., Hercules, CA, USA). Over the course of the bacterial studies, the EC culture in NB was passaged at regular intervals to retain bacterial viability and activity. Additionally, culture studies were conducted in a sterile, laminar flow hood cabinet, and all materials used in culture preparation were handled aseptically. To obtain the EC cells for use in experiments, pellets were obtained from the NB medium by centrifugation at 5000 rpm, after which they were transferred to 0.01 M PBS solutions (pH ∼ 7.3) at the desired final EC concentrations.

EC Biosensing performance of the QD-Apt@cDNA-MB system was assessed by measuring luminescence values before and after incubation with the EC at serial concentrations. Typically, following incubation of the QD-Apt@cDNA-MB with the EC solution (or blank PBS), a magnet was used to extract leftover QD-Apt@cDNA-MB conjugates, which were then excited with the He–Cd laser (325 nm; 2 mW); emission spectra were recorded by the Ocean Optics spectrometer; and the ∼600 nm peak height was extracted and computed as a measure of the luminescent intensity.
